# Effect of Xingnaojing injection for the treatment of acute alcoholism: A protocol of systematic review and meta-analysis

**DOI:** 10.1097/MD.0000000000020785

**Published:** 2020-06-26

**Authors:** Xian Wu, Li-jie Yang, Peng Gao, Zhi-li Qiao, Dan Xu, Fu-hua Zhang

**Affiliations:** a Department of Emergency; b Department of General Medicine; c Department of Neurology, Affiliated Hongqi Hospital of Mudanjiang Medical University, Mudanjiang, China.

**Keywords:** acute alcoholism, effect, safety, Xingnaojing injection

## Abstract

**Background:**

This study will assess the effect of Xingnaojing injection (XNJI) for the treatment of acute alcoholism (AAH).

**Methods:**

The bibliographic literature sources will be systematically searched in MEDLINE, EMBASE, Cochrane Library, China National Knowledge Infrastructure Database, Wan fang Database, and VIP Science Technology Periodical Database. All above electronic databases will be sought from inception to the April 1, 2020. We will not apply any limitations to language and publication time. In addition, we will also search other literature sources. Two reviewers will carry out study selection, data extraction, and methodological quality evaluation, respectively. Any divergences will be resolved by a third reviewer through discussion. We will use RevMan 5.3 software to analyze data analysis.

**Results:**

This study will summarize present evidence to assess the effect of XNJI for the treatment of AAH.

**Conclusion:**

This study will investigate whether XNJI is effective and safety for AAH.

**Systematic review registration:**

INPLASY202040197.

## 1 Introduction

Acute alcoholism (AAH) is one of the most conditions in the emergency admissions,^[1,2]^ which accounts for >21% of all emergency visits.^[3]^ It is characterized by the excessive alcohol drinking that inhibits respiration and heartbeat,^[4–7]^ and it manifests as nausea, vomiting, dizziness, delirium, respiratory failure, coma, and even deaths.^[8–10]^

Xingnaojing injection (XNJI) is widely used to treat AAH during the past few decades in China.^[11]^ Although a recent systematic has published to assess the effectiveness and safety of XNJI for the treatment of AAH in 2019,^[12–28]^ there are still many high-quality randomized controlled trials (RCTs) that have been published after it.^[11]^ Therefore, it is still necessary to perform an update systematic review, and this study will be undertaken based on the most recent high-quality RCTs.

## 2 Methods

### 2.1 Study registration

We have registered on INPLASY202040197. We report this study according to the guideline of Preferred Reporting Items for Systematic Reviews and Meta-analyses Protocols (PRISMA-P).^[29]^

### 2.2 Eligibility criteria for study selection

#### 2.2.1 Type of study

This study will include RCTs that explored the effect of XNJI for the treatment of AAH. We will exclude other studies, such as animal studies, observational studies, review, and uncontrolled studies.

#### 2.2.2 Type of patients

All patients who were diagnosed as AAH will be included in this study, irrespective age, race, sex, and nationality.

#### 2.2.3 Type of interventions

##### 2.2.3.1 Experimental intervention

All patients underwent XNJI for the treatment of AAH will be included in this study.

##### 2.2.3.2 Control intervention

All patients received any management for the treatment of AAH will be considered for inclusion. However, any studies involved XNJI as a control therapy will be excluded.

#### 2.2.4 Types of outcome measurements

Primary outcomes include time to wake up, time to symptoms disappeared, time to cognitive function recovery, and time to motor function recovery.

Secondary outcomes consist of Glasgow coma score, neurological deficit score, emergency observation time, time to return normal systolic blood pressure, time to recover normal breathing, time to recover normal temperature, serum interleukin-6, tumor necrosis factor- α, C-reactive protein, and incidence of adverse reactions.

### 2.3 Search methods for identification of studies

#### 2.3.1 Electronic databases

All potential studies will be sought in electronic databases (MEDLINE, EMBASE, Cochrane Library, China National Knowledge Infrastructure Database, Wan fang Database, and VIP Science Technology Periodical Database) from inception to the April 1, 2020. There are no limitations related to the language and publication time. The search strategy sample for MEDLINE will be showed in Table 1. Similar search strategy sample for other electronic databases will be adapted.

**Table 1:**
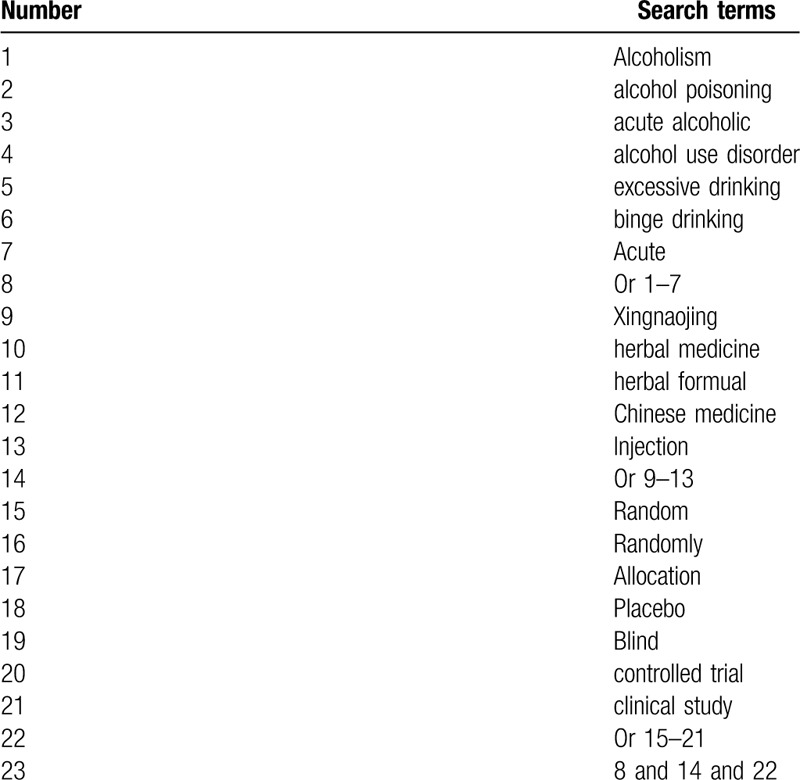
Search strategy for MEDLINE.

#### 2.3.2 Searching other resources

This study will also search other resources, such as thesis, dissertations, conference papers, and reference lists of related reviews.

### 2.4 Data collection and analysis

#### 2.4.1 Study selection

Two reviewers will independently scan the titles and abstracts of all retrieved records, and will eliminate all duplications and irreverent studies. Then, full manuscripts of all remaining trials will be cautiously checked based on the eligibility criteria. All excluded studies with specific reasons will be listed. If conflicts occur between 2 reviewers, we will invite a third reviewer to settle them through consultation. The process of trial literature selection is presented in a PRISMA-P flow chart.

#### 2.4.2 Data extraction and management

Two reviewers will independently extract data using a predefined data extraction form. Any divisions between 2 reviewers will be solved by a third reviewer through discussion. The extracted information consists of study information (eg, title, primary authors, time of publication), patient information (eg, sex, age, diagnostic criteria, and eligibility criteria), study methods, treatment and control specifics, outcome indicators, results, findings, and conflict of interest. Any insufficient or missing data will be required from original trial authors by email, fax, or telephone.

#### 2.4.3 Assessment of risk of bias

Two reviewers will independently assess the risk of bias for each trial using Cochrane risk of bias tool. This tool covers 7 domains and each aspect is further rated as high, unclear, or low risk of bias. Discrepancies between 2 reviewers will be solved through discussion with the help of a third reviewer.

#### 2.4.4 Detection of treatment effect

We will estimate treatment effect of continuous data as mean difference or standardized mean difference and 95% confidence intervals (CIs). The treatment effect of dichotomous data will be presented as risk ratio and 95% CIs.

#### 2.4.5 Assessment of heterogeneity

We will employ *I*^2^ statistics to detect statistical heterogeneity across included trials. *I*^2^ ≤50% indicates acceptable heterogeneity, and we will use a fixed-effects model. *I*^2^ >50% means obvious heterogeneity, and we will utilize a random-effects model.

#### 2.4.6 Subgroup analysis

We will carry out a subgroup analysis to examine obvious heterogeneity according to the different types of study characteristics, details of treatments and comparators, and outcome indicators.

#### 2.4.7 Sensitivity analysis

We will undertake a sensitivity analysis to examine the robustness of merged outcome results by removing trials with low quality.

#### 2.4.8 Publication bias

We will use a Funnel plot and Egger regression test^[30]^ to detect publication bias if >10 eligible trials are included in this study.

#### 2.4.9 Data synthesis

This study will employ RevMan 5.3 software for statistical analysis. If the trials are homogeneous and the data are similar and synthesizable, we will conduct a meta-analysis according to the study information, patient characteristics, details of interventions and controls, and outcome indicators. However, if we detect obvious heterogeneity, we will carry out a subgroup analysis and meta-regression test to investigate the sources of remarkable heterogeneity.

### 2.5 Dissemination and ethics

This study will not need ethical approval, as no individual data will be analyzed. We will publish this study in a peer-reviewed journal.

## 3 Discussion

AAH is a very common disease in the emergency admissions.^[1,2]^ A variety of studies reported that XNJI can be used for the treatment of patients with AAH. However, its effect is still unclear at literature level. Despite a previous study has explored it,^[11]^ a numerous high-quality clinical trials have been published after that.^[12–28]^ Thus, this study will summarize the most recent studies to further investigate the effect and safety of XNJI for the treatment of AAH. Its results may benefit both patients and clinicians.

## Author contributions

**Conceptualization:** Xian Wu, Li-jie Yang, Dan Xu, Fu-hua Zhang.

**Data curation:** Xian Wu, Dan Xu, Fu-hua Zhang.

**Formal analysis:** Xian Wu, Li-jie Yang, Dan Xu, Fu-hua Zhang.

**Investigation:** Fu-hua Zhang.

**Methodology:** Li-jie Yang, Peng Gao, Dan Xu.

**Project administration:** Fu-hua Zhang.

**Resources:** Xian Wu, Li-jie Yang, Peng Gao, Zhi-li Qiao, Dan Xu.

**Software:** Xian Wu, Li-jie Yang, Peng Gao, Zhi-li Qiao.

**Supervision:** Fu-hua Zhang.

**Validation:** Xian Wu, Li-jie Yang, Zhi-li Qiao, Dan Xu, Fu-hua Zhang.

**Visualization:** Xian Wu, Li-jie Yang, Peng Gao, Zhi-li Qiao, Dan Xu, Fu-hua Zhang.

**Writing – original draft:** Xian Wu, Li-jie Yang, Peng Gao, Zhi-li Qiao, Dan Xu, Fu-hua Zhang.

**Writing – review & editing:** Xian Wu, Peng Gao, Zhi-li Qiao, Fu-hua Zhang.
